# Supplementary material for: Immunohistochemical comparison of three programmed death-ligand 1 (PD-L1) assays in triple-negative breast cancer
Source: PLoS One. 2021 Sep 24;16(9):e0257860. doi: 10.1371/journal.pone.0257860 (PMC8462691; doi:10.1371/journal.pone.0257860)
Supplement: S1 File — (PDF) [file pone.0257860.s001.pdf]

No.	Age	menoposal status	pathological maximum invasive compornent (mm)	positive lymph nodes	pTNM	pStage	histological grading	ly	v	Stromal TILs	Ki-67(Operation: %)	Adjuvant chemotherapy	relapse	RFS (months)	dead	OS(months)
5	75	-	15	0	T1cN0M0	I	3	+	-	-	40	+	-	20	-	20
7	31	+	12	0	T1cN0M0	I	3	-	-	+	70	+	-	20	-	20
15	41	+	20	0	T1cN0M0	I	3	-	+	-	40	+	-	35	-	35
16	68	-	18	0	T1cN0M0	I	3	-	-	+	70	+	-	36	-	36
21	66	-	22	0	T2N0M0	II A	2	+	-	-	20	+	-	31	-	31
42	83	-	18	2	T1cN1M0	II A	3	-	-	-	70	-	-	18	-	18
44	76	-	18	0	T1cN0M0	I	2	-	-	+	40	-	-	40	-	40
57	77	-	6	0	T1bN0M0	I	1	-	-	-	15	+	-	34	+	34
59	65	-	10	2	T1bN1M0	II A	2	+	-	+	25	+	-	53	-	53
62	77	-	51	0	T3N0M0	II B	3	+	+	-	80	-	+	11	-	23
63	68	-	20	0	T1cN0M0	I	2	+	-	-	45	-	-	60	-	60
65	62	-	28	0	T2N0M0	II A	2	+	-	+	38	+	-	60	-	60
66	74	-	35	9	T2N2M0	III A	2	+	+	+	50	-	+	18	+	36
67	54	-	22	0	T2N0M0	II A	2	+	+	-	15	+	-	60	-	60
70	80	-	12	0	T1bN0M0	I	2	+	+	-	20	-	-	60	-	60
72	93	-	25	2	T2N1M0	II B	2	+	+	-	40	-	-	60	-	60
73	60	-	42	0	T2N0M0	II A	2	+	+	+	80	+	-	60	-	60
76	87	-	55	not tested	T4a cN0M0	III B	3	+	+	-	40	-	-	22	+	29
79	73	-	38	0	T2N0M0	II A	3	+	+	-	55	unknown	-	44	-	44
81	84	-	40	0	T2N0M0	II A	3	+	+	-	80	-	-	33	-	60
85	45	unknown	20	0	T1cN0M0	I	3	+	+	-	70	+	-	11	-	11
86	67	-	20	0	T1cN0M0	I	3	+	+	-	50	+	-	60	-	60
87	71	-	16	1	T1cN1M0	II A	2	+	-	-	70	-	+	9	+	18
88	70	-	12	0	T1cN0M0	I	2	+	-	+	28	-	-	60	-	60
89	60	-	13	not tested	T1c cN0M0	I	2	+	-	-	80	-	-	60	-	60
90	88	-	40	2	T2N1M0	II B	2	+	+	-	50	-	+	22	-	23
91	58	-	20	not tested	T1c cN0M0	I	3	+	+	+	50	+	-	60	-	60
93	44	+	27	3	T2N1M0	II B	3	+	+	-	47	+	-	50	-	50
94	69	-	54	0	T4bN0M0	III B	2	+	-	-	40	unknown	-	60	-	60
97	70	-	10	0	T1bN0M0	I	1	+	-	-	25	+	-	60	-	60
99	37	+	52	2	T3N1M0	III A	3	+	+	-	80	+	+	9	+	16
100	80	-	21	0	T2N0M0	II A	3	+	+	-	80	+	-	60	-	60
102	61	-	40	5	T2N2M0	III A	3	+	+	-	25	+	-	48	-	60
103	37	+	30	0	T2N0M0	II A	3	+	+	-	40	+	-	52	-	52
110	74	-	25	0	T2N0M0	II A	2	+	+	+	40	+	-	60	-	60
111	78	-	40	3	T2N1M0	II B	3	+	+	-	49	+	-	60	-	60
118	89	-	35	not tested	T2 cN0M0	II A	3	+	+	-	5	-	-	46	-	46
119	67	-	7	not tested	T1b cN0M0	I	2	+	-	-	60	+	-	60	-	60
125	60	-	6	not tested	T1b cN0M0	I	2	-	-	-	Not evaluated	-	-	60	-	60
127	55	-	50	0	T2N0M0	II A	3	+	+	-	15	unknown	-	60	-	60
129	44	+	30	not tested	T2 cN0M0	II A	3	+	-	+	30	+	-	60	-	60
131	36	+	13	0	T1cN0M0	I	2	+	-	-	70	+	-	60	-	60
138	62	-	26	0	T2N0M0	II A	2	+	+	+	10	+	-	60	-	60
140	67	-	25	0	T2N0M0	II A	2	+	+	-	10	+	-	60	-	60
141	81	-	18	2	T1cN1M0	II A	2	+	+	-	15	+	-	60	-	60
143	82	-	12	not tested	T1c cN0M0	I	3	+	+	-	30	-	-	31	-	31
144	74	-	23	not tested	T2 cN0M0	II A	2	+	-	-	70	-	-	60	-	60
145	62	-	2	not tested	T1a cN0M0	I	2	-	-	+	Not evaluated	-	-	60	-	60
147	80	-	40	1	T4bN1M0	III B	3	+	+	-	30	-	-	56	+	56
148	64	-	35	4	T2N2M0	III A	2	+	+	-	15	+	+	40	-	60
149	69	-	25	0	T2N0M0	II A	3	+	+	+	25	+	-	60	-	60
151	79	-	22	not tested	T2 cN0M0	II A	3	+	+	-	50	-	-	60	+	60
152	54	-	18	0	T1cN0M0	I	3	+	+	-	60	+	-	60	-	60
153	58	-	20	not tested	T1c cN0M0	I	3	+	+	-	15	+	+	35	+	42
156	50	-	20	18	T1cN3M0	III C	3	+	+	+	70	-	+	11	+	41
157	41	+	17	not tested	T1c cN0M0	I	3	+	+	+	60	+	-	60	-	60
159	58	-	2	0	T1aN0M0	I	3	+	-	+	Not evaluated	+	-	48	-	48
161	52	-	20	0	T1cN0M0	I	3	+	+	+	80	+	-	60	-	60
162	45	+	25	0	T2N0M0	II A	3	+	+	-	Not evaluated	-	+	15	+	57
163	71	-	12	not tested	T1c cN0M0	I	2	+	-	-	60	-	-	53	-	53
165	69	-	7	not tested	T1b cN0M0	I	2	+	-	-	80	-	-	60	-	60
168	81	-	40	0	T2N0M0	II A	2	-	-	+	15	-	-	16	-	16
